# Cloning of *pcB* and *pcA* Gene from *Gracilariopsis lemaneiformis* and Expression of a Fluorescent Phycocyanin in Heterologous Host

**DOI:** 10.3390/genes10050322

**Published:** 2019-04-26

**Authors:** Deguang Sun, Xiaonan Zang, Yalin Guo, Dongfang Xiao, Xuexue Cao, Zhu Liu, Feng Zhang, Yuming Jin, Jiawei Shi, Zhendong Wang, Rui Li, Zhaxi Yangzong

**Affiliations:** Key Laboratory of Marine Genetics and Breeding, Ministry of Education, Ocean University of China, Qingdao 266003, Shandong, China; sundeguang@stu.ouc.edu.cn (D.S.); 17669475369@163.com (Y.G.); 18753364418@163.com (D.X.); caoxuex@126.com (X.C.); liuzhu@stu.ouc.edu.cn (Z.L.); zfhhxxttxs@163.com (F.Z.); happycyz@126.com (Y.J.); shijiawei@stu.ouc.edu.cn (J.S.); zhendongya123@163.com (Z.W.); LRlirui0611@163.com (R.L.); zxyz1773494902@163.com (Z.Y.)

**Keywords:** *Gracilariopsis lemaneiformis*, phycocyanin, fluorescence, cloning, expression

## Abstract

In order to study the assembly mechanism of phycocyanin in red algae, the apo-phycocyanin genes (*pcB* and *pcA*) were cloned from *Gracilariopsis lemaneiformis*. The full length of phycocyanin β-subunit (*pc*B) contained 519 nucleotides encoding a protein of 172 amino acids, and the full length of phycocyanin α-subunit(*pc*A) contained 489 nucleotides encoding a protein of 162 amino acids. Expression vector pACYCDuet-*pcB*-*pcA* was constructed and transformed into *E. coli* BL21 with pET-*ho*-*pcyA* (containing *ho* and *pcyA* gene to synthesize phycocyanobilin). The recombinant strain showed fluorescence activity, indicating the expression of optically active phycocyanin in *E. coli*. To further investigate the possible binding sites between phycocyanobilin and apo-phycocyanin, Cys-82 and Cys-153 of the β subunit and the Cys-84 of the α subunit were respectively mutated, and four mutants were obtained. All mutant strains had lower fluorescence intensity than the non-mutant strains, which indicated that these mutation sites could be the active binding sites between apo-phycocyanin and phycocyanobilin (PCB). This research provides a supplement for the comprehensive understanding of the assembly mechanism of optically active phycocyanin in red algae.

## 1. Introduction

Phycobilisomes (PBSs) are a complex light-harvesting protein mainly found in cyanobacteria and red algae, which capture light energy in a spectrum range of 480 to 650 nm and transmit it to the photosystem II [1,2]. PBSs consist of two parts, including brightly colored phycobiliproteins (PBPs) and non-pigmented linker proteins. The colored phycobiliproteins (PBPs) are responsible for the absorption and transmission of light energy. The function of the non-pigmented linker protein is to guide the assembly of PBSs [2,3]. PBP can be used in many fields, not only as a fluorescent probe or food additive, but also as an anti-oxidation, anti-tumor, and anti-inflammatory agent [4,5,6]. PBP is normally composed of phycoerythrin (PE), phycocyanin (PC), and allophycocyanin (APC).

Phycocyanin (PC) consists of two subunits (α and β), and each subunit is covalently bonded 1 to 4 phycocyanobilin (PCB) under the catalysis of chromophore lyase [7,8,9,10]. Phycocyanobilin is a linear tetrapyrrole prosthetic group that is covalently bonded to a specific cysteine residue through a thioether bond, which gives PBPs a brilliant color and light absorption properties [11,12]. The biosynthesis of phycocyanobilin begins with heme and then proceeds through reduction and isomerization steps in the catalytic reaction of heme oxygenase (*ho*) and ferredoxin oxidoreductase (*pcyA*) [13]. Apo-phycocyanin is linked with PCB at a specific binding site. According to reports, the Cys-84 of α subunit (α-PC,CpcA) is linked to a PCB, the Cys-82 and Cys-153 of β subunit (β-PC, CpcB) are each combined with a PCB [14,15,16].

To study the biosynthetic pathway of phycocyanin with optical activity, an optically active phycocyanin of *Synechocystis* sp. PCC6803 has been expressed in a heterologous host *E. coli* BL21 [15,17]. Co-expression of phycocyanin alpha subunit gene (*cpcA*), heme oxygenase gene (*ho*), PCB–ferredoxin oxidoreductase gene (*pcyA*), and chromophore lyase genes(*cpcE/F*) in *E. coli* BL21 obtain a fluorescently active phycocyanin α subunit, which demonstrates that the assembly mechanism of phycocyanin can be studied in a heterogenic host [10,18,19]. The apo-phycocyanin alpha and beta subunit genes *cpcA* and *cpcB* in the *Arthrospira platensis* FACHB314 have been cloned in our laboratory and co-transformed with heme oxidase gene (*ho*), PCB–ferredoxin oxidoreductase gene (*pcyA*), α-subunit chromozyme genes *cpcE/F*, and β-Subunit chromozyme genes *cpcU, cpcS,* and *cpcT* into *E. coli* BL21 to express phycocyanin with optical activity [16,20,21].

In recent years, many achievements have been made in the field of gene engineering on the expression of optically active phycocyanin [15,17]. Because the transgenic technology of prokaryotic cyanobacteria is relatively mature, and the whole genome sequencing of several cyanobacteria has been completed. Therefore, current research regarding phycocyanin mainly concentrates on cyanobacteria, whereas the synthesis of phycocyanin in red algae is rarely studied. The phycobilisomes are usually composed of phycoerythrin (PE), phycocyanin (PC) and allophycocyanin (APC) [3,11]. Some cyanobacteria may not contain phycoerythrin, while red algae contain complete phycobilisome, including these three phycobiliproteins. Therefore, studying the synthesis of phycocyanin in red algae is of great significance for studying the overall assembly of phycobilisomes. In this experiment, the apo-phycocyanin genes (*pcB* and *pcA*) in *Gracilariopsis lemaneiformis* were first cloned and co-transformed with pET24a-*ho-pcyA*, containing heme oxygenase gene (*ho*), ferredoxin oxidoreductase gene (*pcyA*), into *E. coli* BL21 to express phycocyanin with optical activity. In order to detect the binding sites of PCB and apo-phycocyanin, the Cys-84 of α subunit and Cys-82 and Cys-153 of β subunit were mutated to alanine (Ala) with different properties, and four mutant expression vectors were constructed: pACYCDuet-*pcB* (C82A), pACYCDuet-*pcB* (C153A), pACYCDuet-*pcB* (C82A, C153A), and pACYCDuet-*pcA* (C84A). Each of these four vectors was co-transformed with pET24a-*ho-pcyA* into *E. coli* BL21. Sodium dodecyl sulfate polyacrylamide gel electrophoresis (SDS-PAGE) and Western blotting were used to detect the expression of phycocyanin, and the fluorescent emission spectrum of the recombinant expression strain was determined by fluorescence spectrophotometer. This study lays a foundation for understanding the assembly mechanism of phycocyanin in red algae, providing an experimental basis for synthesis of optically active phycocyanin of red algae in heterologous hosts.

## 2. Materials and Methods

### 2.1. Strains and Plasmids

**Figure 1:**
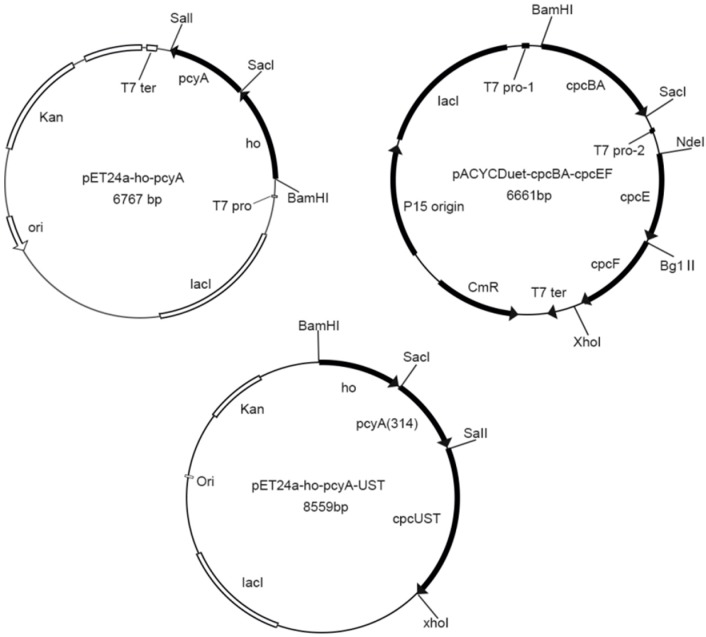
Plasmids of pET24a-*ho*-*pcyA*, pACYCDuet-*cpcBA*-*cpcEF* and pET24a-*ho*-*pcyA*-UST.

The *G. lemaneiformis* used in this study was collected at Fushan Bay, Qingdao (E120.358, N:36.065) and cultured in filtered seawater at 23 °C in laboratory. Plasmids pET24a-*ho*-*pcyA,* pACYCDuet-*cpcBA*-*cpcEF*, and pET24a-*ho*-*pcyA*-UST were constructed previously in our laboratory (Figure 1) [21]. The plasmid pET24a-*ho*-*pcyA* contained the genes *ho* and *pcyA* encoding the heme oxygenase gene and ferredoxin oxidoreductase in *A. platensis* respectively. The plasmid pACYCDuet-*cpcBA*-*cpcEF* contained the genes *cpcB* and *cpcA* encoding apo-phycocyanin β-subunit and α-subunit, and the genes *cpcE* and *cpcF* encoding the chromophorelyase in *A. platensis*. The plasmidpET24a-*ho*-*pcyA*-UST contains the genes *ho* and *pcyA* encoding the heme oxygenase gene and ferredoxin oxidoreductase in *A.platensis,* and the genes *cpcU*, *cpcS* and *cpcT* encoding the chromophore lyase in *A. platensis*. Table 1 shows all the strains and plasmids in this study.

### 2.2. Genomic DNA Extraction and Gene Cloning of pcB and pcA

Genomic DNA of *G. lemaneiformis* was extracted by using the Universal Genomic DNA Extraction kit (TaKaRa, China). Primers were designed based on the gene sequence from the transcriptome data of the *G. lemaneiformis* in our laboratory [22]. According to the gene sequence, the *pcB* gene and the *pcA* gene are adjacent to each other in the genome. By using the upstream primer of *pcB* and the downstream primer of *pcA*, a gene fragment containing both genes of *pcB* and *pcA* was cloned and named as gene *pcBA*. All specific primers are listed in Table 2. To ligate the cloned fragment to the expression vector, restriction enzyme sites *Bam*HI and *Sac*I were added to the primers in advance (the sites of restriction enzyme are underlined). By using the *G. lemaneiformis* genome DNA as the template, the genes *pcB*, *pcA* and *pcBA* were amplified by PCR. The PCR product was gel purified and ligated into the pEASY-T3 vector for sequencing (Sangon Biotech, Shanghai, China). After sequenced and compared to the sequences in the GenBank, the PCR products were identified as genes *pcB*, *pcA* and *pcBA*. The PCR amplification has three stages: 5 min at 94 °C, followed by 40 rounds of 30 s at 94 °C, 30 s at 55 °C (annealing temperature reference primer table) and 1 min at 72 °C, and extended at 72 °C for 10 min.

Analysis of genes according to the method of Zhang et al. [16]. Gene *pcB* and *pcA* had homologous analysis performed using the BLAST program (http://www.ncbi.nlm.nih.gov/BLAST/) based on the GenBank nucleic acid database [23]. The amino acid sequences of the genes *pcB* and *pcA* were translated using DNAMAN software [16]. Molecular weight and theoretical isoelectric point were predicted by using the ExPASy ProtParam (http://us.expasy.org/tools/protparam.html). Secondary structure was predicted by NPS@: GOR4 secondary structure prediction. A phylogenetic tree was constructed by using MEGA 7.0 software. Boot execution was performed 1000 times to get the support value for each branch [24].

### 2.3. Vector Construction

The *pcBA*, *pcB*, *pcA* gene were digested with restriction enzymes *Bam*HI and *Sac*I, and the obtained gene fragment *pcBA*, *pcB*, *pcA* were connected to the vector pACYCDuet-1 to obtain the recombinant plasmids pACYCDuet-*pcBA*, pACYCDuet-*pcB*, pACYCDuet-*pcA*. The plasmid pACYCDuet-*cpcBA*-*cpcEF* was constructed by our laboratory and extracted by using a high purity plasmid extraction kit (Biomed, Beijing, China). After digestion by the restriction enzymes *Bam*HI and *Sac*I, the gene *cpcBA* in the plasmid pACYCDuet-*cpcBA*-*cpcEF* was replaced with the gene *pcBA* to obtain recombinant plasmid pACYCDuet-*pcBA*-*cpcEF*.

In order to explore the site of apo-phycocyanin to bind PCB, Cys-82 and Cys-153 of the β subunit and Cys-84 of the α subunit were mutated to Ala because of the different properties between these two amino acids. Ala’s preferred codon in *G. lemaneiformis* is GCT. Designed primers for mutation are shown in Table 3. Vectors pACYCDuet-*pcB* and pACYCDuet-*pcA* were taken as templates, and the site-directed mutant linear DNA was amplified by PCR using the Mutation Kit (TaKaRa, Dalian, China). Then linear DNA was linked by using the DNA Blunting Kit (TaKaRa, Dalian, China). All plasmids were transformed into *E. coli* DH5α and verified by sequencing. Therefore, the mutated vectors pACYCDuet-*pcB* (C82A), pACYCDuet-*pcB* (C153A) and pACYCDuet-*pcA* (C84A) were obtained. Then plasmid pACYCDuet-*pcB* (C82A) was taken as a template, vector pACYCDuet-*pcB* (C82A, C153A) containing two mutation sites was obtained through the same method.

### 2.4. Plasmid Transformation and Protein Expression

Seven recombinant expression plasmids pACYCDuet-*pcBA*, pACYCDuet-*pcB*, pACYCDuet-*pcA*, pACYCDuet-*pcB* (C82A), pACYCDuet-*pcB* (C153A), pACYCDuet-*pcB* (C82A, C153A), pACYCDuet-*pcA* (C84A) were separately transformed into *E. coli* BL21 with the plasmid pET24a-*ho*-*pcyA*. The recombinant plasmids pET24a-*ho*-*pcyA*-UST and pACYCDuet-*pcBA*-EF were co-transformed into *E. coli* BL21. An LB medium plate containing 34 μg·mL^−1^ of chloramphenicol (Cm) and 100 μg·mL^−1^ of kanamycin (Km) was prepared. The positive colonies were screened on the plate and the inserted genes was confirmed by PCR amplification and sequencing.

To express the recombinant protein in the transformed strain, 1 mL of overnight culture solution of the recombinant strain was added to 100 ml of lysogeny broth (LB) medium containing the antibiotics Cm and Km, and cultured at 37 °C until the optical density (OD_600_) was 0.5–0.6. 0.1 mM isopropyl-β-D-thiogalactoside (IPTG) was added in the LB medium to induce the expression of recombinant protein. The cells were incubated for 4 h at 200 rpm. OD_600_ was measured again and calculated to ensure the same cell density for all samples. 2 mL of the cell broth was centrifuged and suspended in 400 μL of 0.01 M PBS (phosphate buffered saline) buffer (pH 7.2). The resuspending solution was added 100 μL of the loading buffer and then boiled for 5 min to prepare for SDS-PAGE and Western blotting. The remaining cell broth was collected by centrifugation at 4000× *g* for 20 min. The cell pellet was washed with 0.01 M PBS buffer (pH 7.2) and resuspended in 4 mL of PBS buffer. Then cell suspension was ultrasonic disrupted on ice for 6 min. After the cell suspension was centrifuged at 12,000× *g* for 15 min, the fluorescence emission spectrum of the supernatant was detected.

### 2.5. Recombinant Protein Analysis

The protein samples were analyzed by SDS polyacrylamide gel electrophoresis [25]. SDS-PAGE gels were prepared using a 5% stacking gel and a 15% separation gel (MDBio, Taiwan, China). The proteins were visualized by staining with Coomassie Blue R-250 (Sangon Biotech, Qingdao, China).

Western blot analysis was performed using Rb c-phycocyanin (Maded by Boster Biological Technology co.ltd, Qingdao, China) and peroxidase-conjugated goat anti-rabbit IgG (Sangon Biotech, Qingdao, China) as primary and secondary antibodies, and the dilution factors were 1:1000 (primary antibodies) and 1:2000 (secondary antibodies). The expression of phycocyanin was judged by observing whether there is a hybridization strip in the nitrocellulose membrane.

### 2.6. Fluorescence Emission Spectra Analysis

Fluorescence spectrophotometer (HITACHIF-4600) was used for detect the fluorescence emission spectrum excited at 580 nm [26]. The parameters were set to an excitation and emission slit width of 10 nm and a scanning speed of 1200 nm/min. The fluorescence intensity and wavelength of the highest emission peak were recorded [27]. All the experiments were repeated three times.

## 3. Results

### 3.1. Analysis of the Genes pcB and pcA

**Figure 2:**
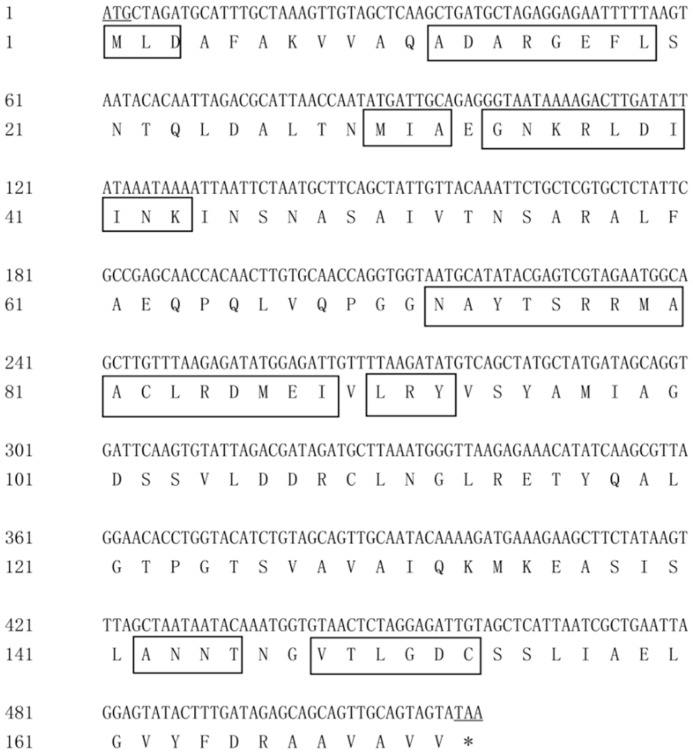
DNA and amino acid sequences of *pcB*. Underline indicates start or stop codons, and boxes indicate conserved sites.

The cloned genes *pcB*, *pcA* and *pcBA* were sequenced for three clones respectively to prove that the gene sequences were correct. The DNA and deduced amino acid sequence of the gene *pcB* are shown in Figure 2. The DNA sequence of *pcB* consisted of 519 nucleotides and the GC content (guanine-cytosine content) was 51.5%. The open reading frame (ORF) started from ATG and ended at TAA, which encoded a protein of 172 amino acids. The predicted molecular weight was 18.3 kDa, and the theoretical isoelectric point of the protein was 5.13.

**Figure 3:**
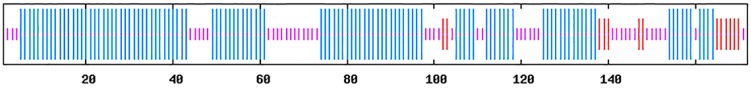
The secondary structure of PcB. Long vertical bars are α-helix, medium vertical bars are extended strands, and short vertical bars are random coil regions.

The secondary structure prediction of PcB is shown in Figure 3. The α-helix region accounted for 64.53% of the protein, which was distributed throughout the peptide chain and was more concentrated in the C-terminal region. The extended chain accounted for 7.56%, which was mainly in the N-terminal region. The random coiled region accounted for 27.91% and was dispersed throughout the protein.

**Figure 4:**
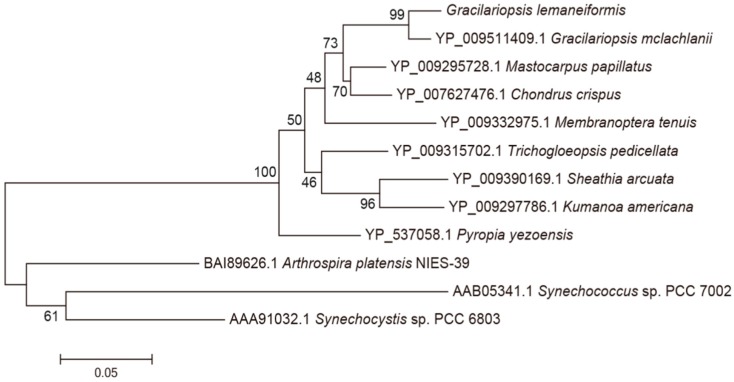
Phylogenetic analysis of PcB amino acid sequences. Numbers at the nodes represent the bootstrap values. The evolutionary distance between the groups is indicated by the scale (0.05 = 5% difference). The sequences of PcB taken from GenBank are as follows: *Gracilariopsis mclachlanii* (YP009511409.1), *Pyropia yezoensis* (YP537058.1), *Mastocarpus papillatus* (YP009295728.1 ), *Chondrus crispus* (YP007627476.1), *Membranoptera tenuis* (YP009332975.1), *Trichogloeopsis pedicellata* (YP009315702.1), *Sheathia arcuate* (YP009390169.1), *Kumanoa Americana* (YP009297786.1), *Arthrospira platensis* NIES-39 (BAI89626.1), *Synechococcus* sp. PCC7002 (AAB05341.1), *Synechocystis* sp. PCC 6803 (AAA91032.1).

BLAST and homology analysis revealed that the PcB of *G. lemaneiformis* had high identity with CpcB family protein and contained the conserved motif of phycocyanin β subunit, including MLD, ADARGEFL, MIA, GNKRLDIINK, NAYTSRRMAACLRDMEI, LRY, ANNT and VTLGDC. In the phylogenetic analysis of PcB (Figure 4), the PcB of *G. lemaneiformis* was clustered with other algae of Gracilariaceae and has the closest relationship with *Gracilariopsis mclachlanii* (YP009511409.1) with a confidence of 99%. Red algae and cyanobacteria are clustered in different branches.

**Figure 5:**
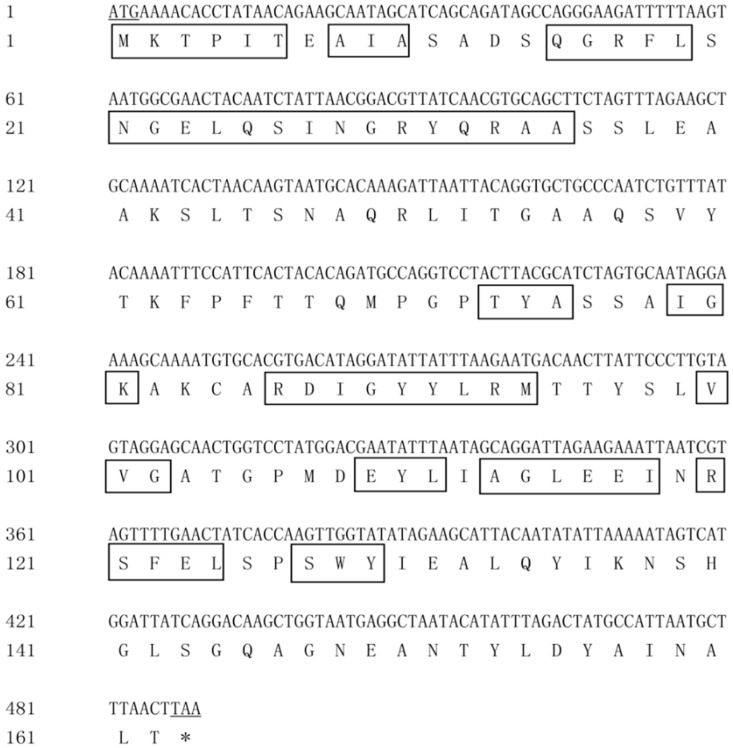
DNA and amino acid sequence of *pcA*. Underline indicates start or stop codons, and boxes indicate conserved sites.

The complete coding sequence and deduced amino acid sequence of *pcA* are shown in Figure 5. The full-length DNA of gene *pcA* consisted of 489 nucleotides and the GC content was 45.8%. The ORF started at ATG and ended at TAA, which encoded a protein of 162 amino acids. The predicted molecular weight was 17.5 kDa, and the theoretical isoelectric point of the protein was 6.57.

**Figure 6:**
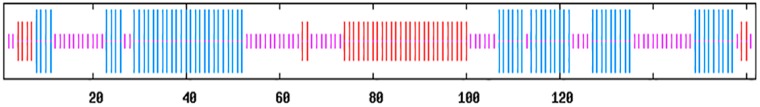
The secondary structure of PcA. Long vertical bars are α-helix, medium vertical bars are extended strands, and short vertical bars are random coil regions.

The secondary structure prediction of PcA is shown in Figure 6. The α-helix region accounted for 40.12% of the protein, which was mainly distributed at both ends of the peptide chain. The extended chain accounted for 21.60%, which was mainly in the middle region of the peptide chain. The random coil region was dispersed throughout the protein, accounting for 38.27%.

**Figure 7:**
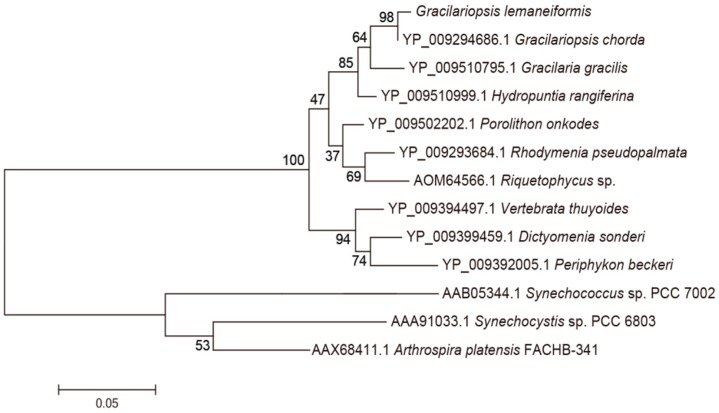
Phylogenetic analysis of PcA amino acid sequences. Numbers at the nodes represent the bootstrap values. The evolutionary distance between the groups is indicated by the scale (0.05 = 5% difference). The sequences of PcA taken from GenBank are as follows: *Gracilariopsis chorda* (YP009294686.1), *Gracilaria gracilis* (YP009510795.1), *Hydropuntia rangiferina* (YP009510999.1), *Porolithon onkodes* (YP009502202.1), *Rhodymenia pseudopalmata* (YP009293684.1), *Riquetophycus* sp. (AOM64566.1), *Dictyomenia sonderi* (YP009399459.1), *Periphykon beckeri* (YP009392005.1), *Vertebrata thuyoides* (YP009394497.1), *Synechocystis* sp. PCC 6803 (AAA91033.1), *Synechococcus* sp. PCC 7002 (AAB05344.1), *Arthrospira platensis* FACHB-341 (AAX68411.1).

BLAST and homology analysis revealed that the PcA of *G. lemaneiformis* had high identity with CpcA family protein and contained the conserved motif of phycocyanin beta subunit, including MKTPIT, AIA, QGRFL, NGELQSINGRYQRAA, TYA, IGK, RDIGYYLRM, VVG, EYL, AGLEEI, RSFELand SWY. In the phylogenetic analysis of PcA (Figure 7), the PcA of *G. lemaneiformis* clustered with *Gracilariopsis chorda* (YP009294686.1) with a confidence of 98%. Red algae clustered into a branch and belonged to different branches away from cyanobacteria. The homology analysis indicated that the sequence similarity of PcB and PcA was consistent with the affinity of species.

### 3.2. Construction of Recombinant Strain

Eight expression vectors containing *pcBA, pcBA*-*cpcEF*, *pcB*, *pcB* (C82A), *pcB* (C153A), *pcB* (C82A, C153A), *pcA* and *pcA* (C84A) were constructed respectively. pACYCDuet-*pcBA*, pACYCDuet-*pcB*, pACYCDuet-*pcB* (C82A), pACYCDuet-*pcB* (C153A), pACYCDuet-*pcB* (C82A, C153A), pACYCDuet-*pcA* and pACYCDuet-*pcA* (C84A) were co-transformed with vector pET24a-*ho*-*pcyA* into *E. coli* BL21 respectively. pACYCDuet-*pcBA*-*cpcEF* and pET24a-*ho*-*pcyA*-UST were co-transformed into *E. coli* BL21. pACYCDuet-*1* and pET-24a(+) were co-transformed into *E. coli* BL21 as the control. Through PCR detection and sequencing (Sangon Biotech, Qingdao, China), the inserted gene was confirmed to exist in the recombinant strain and the expression cassette was correct. See Table 4.

### 3.3. Expression of the Recombinant Proteins 

**Figure 8:**
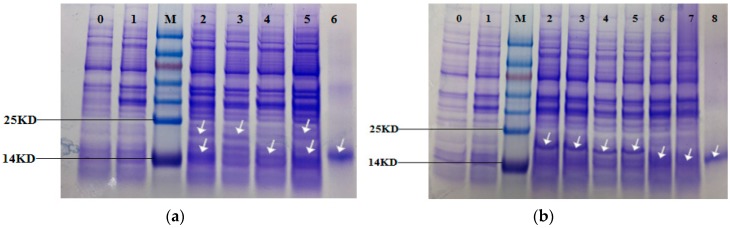
Sodium dodecyl sulfate polyacrylamide gel electrophoresis (SDS-PAGE) of the recombinant strains and mutant strains. Lane 0 is the control strains. Lane 1–6 in (**a**) are HP, BAHP, BHP, AHP, BAEFHPUST and phycocyanin standard. Lane 1–8 in (**b**) are HP, BHP, B(82)HP, B(153)HP, B(82,153)HP, AHP, A(84)HP and phycocyanin standard. The recombinant strains BAHP, BHP, AHP, BAEFHPUST in (**a**) and BHP, B(82)HP, B(153)HP, B(82,153)HP, AHP, A(84)HP in (**b**) all had significant bands (indicated by the arrow) at about 18 kDa, which were identical to the phycocyanin standard. While the control strain and recombinant strain HP only had a very shallow band at this position. In BAHP and BAEFHPUST, two significant bands were detected. The larger one was the β subunit with more amino acids and the smaller one was the α subunit.

**Figure 9:**
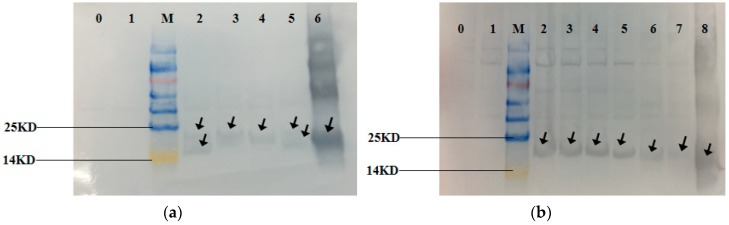
Western Blotting of the recombinant strains and mutant strains. Lane 0 is control strains. Lane 1–6 in (**a**) are HP, BAHP, BHP, AHP, BAEFHPUST and phycocyanin standard. Lane 1–8 in (**b**) are HP, BHP, B(82)HP, B(153)HP, B(82,153)HP, AHP, A(84)HP and phycocyanin standard. The recombinant strains BAHP, BHP, AHP, BAEFHPUST, HP, BHP, B(82)HP, B(153)HP, B(82,153)HP, AHP, A(84)HP and phycocyanin standard showed obvious hybridization bands at around 18 kDa, which proved that phycocyanin was expressed. There was no hybridization band in control strains and HP strains. Two hybridization bands were detected in BAHP and BAEFHPUST. The larger one was the β subunit with more amino acids and the smaller one was the α subunit.

The expression of the recombinant protein was induced in *E. coli* BL21 cells by IPTG (Isopropyl β-D-1-thiogalactopyranoside)and the recombinant strains showed different degrees of blue-green color compared to the off-white control (Control). The SDS-PAGE gel (Figure 8) showed that the recombinant strains BAHP, BHP, AHP, BAEFHPUST, BHP, B(82)HP, B(153)HP, B(82,153)HP, AHP and A(84)HP all had more distinct bands at about 18 kDa compared to the control strain and the recombinant strain HP. Western blot (Figure 9) showed that blank control strains and recombinant strains HP had no hybridization bands, while the recombinant strains BAHP, BHP, AHP, BAEFHPUST and mutant strain B (82) HP, B (153) HP, B (82, 153) HP, A(84) HP all had an immunoreactive band of approximately 18 kDa, indicating that phycocyanin was expressed in *E. coli* BL21. β subunit had more amino acids than α subunit, so its band size was larger than that of α subunit. In the recombinant strains BAHP and BAEFHPUST, two subunits were expressed and there were two hybridization bands were observed.

### 3.4. Fluorescence Emission Spectra

**Figure 10:**
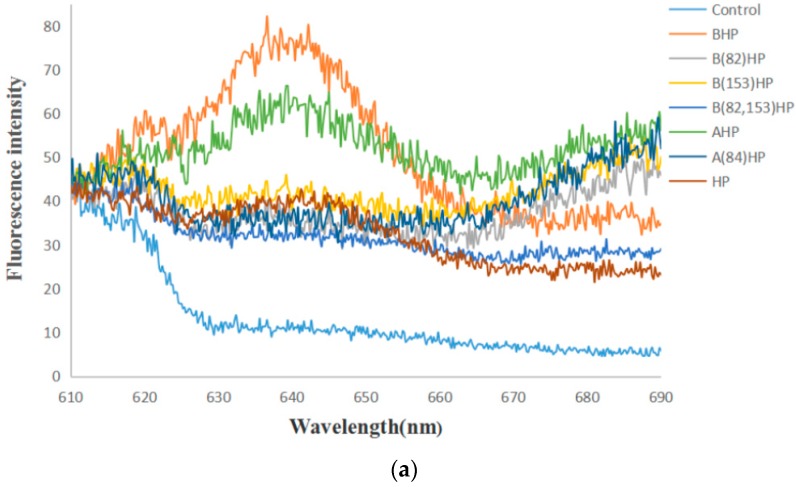

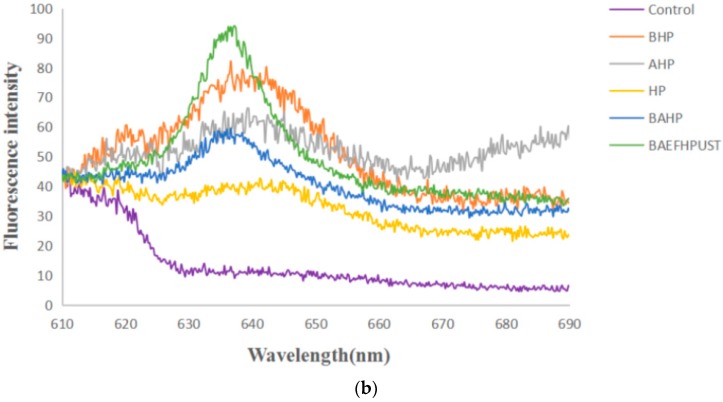
Fluorescence emission spectra of the recombinant strains (HP, BHP, AHP, BAHP, BAEFHPUST) and mutant strains (B(82)HP, B(153)HP, B(82,153)HP, A(84)HP) after excitation at 580 nm. In (**a**), the fluorescent peak of the mutant strains B (153) HP, B (82) HP and B (82, 153) was lower than that of the unmutated strain BHP, and the fluorescent peak of the mutant strain A (84) HP was lower than that of the unmutated strain AHP. In (**b**), the strain BAEFHPUST had the highest fluorescence peak.

A specific PC (phycocyanin) fluorescence emission spectrum after excitation at 580 nm was examined by using cell suspension of the recombinant strain (Figure 10). The results showed that the blank control strains had no fluorescence peak, while recombinant strains BAEFHPUST, BAHP, BHP, AHP, HP, B (82) HP, B (153) HP, B (82, 153) HP, and A (84) HP had varying degrees of fluorescence emission peaks around 640 nm, which proved the successful expression of phycocyanin with fluorescence activity. Among them, the recombinant strain BAEFHPUST had the highest fluorescence intensity, which proved that the presence of chromophore lyase could indeed promote the correct assembly of phycocyanobilin (PCB) and apo-PC. The fluorescence intensity of mutant strains B (82) HP, B (153) HP, B (82, 153) HP, and A (84) HP was lower than that of the strain without mutation. It was indicated that Cys-82 and Cys-153 may have been the active sites that mediated the binding to the PCB on the beta subunit, and Cys-84 may have been the active site on the alpha subunit.

## 4. Discussion

Phycocyanin contributes to many important biological activities, such as anti-oxidation, anti-tumor, or as fluorescent labels and dyes. A number of studies have achieved the heterologous expression of optically active phycocyanin [10,15], but these mainly focused on cyanobacteria rather than red algae [10,15].

In this study, *pcBA* gene was first cloned from *G. lemaneiformis*. The result of sequence alignment shows that PcA and PcB are relatively conserved proteins in algae. PcA has 12 conserved active domains including MKTPIT, AIA, QGRFL, NGELQSINGRYQRAA, TYA, IGK, RDIGYYLRM, VVG, EYL, AGLEEI, RSFEL, and SWY, which have high identity with the CpcA family protein. Likewise, PcB demonstrated high identity with the CpcB family protein, having nine conserved domains including MLD, ADARGEFL, MIA, GNKRLDIINK, NAYTSRRMAACLRDMEI, LRY, ANNT, VTL, and GDC. In order to verify whether the assembly mechanism of phycocyanin in red algae is similar with cyanobacteria, the recombinant strains BHP, AHP, BAHP, and BAEFHPUST were constructed. Whether in cyanobacteria or in heterologous hosts, it has been reported that as long as the PCB is properly attached to apo-PC, it is detectable from the specific fluorescence spectra [10,15]. All recombinant strains in this experiment showed a specific blue-green color. The recombinant strains BAEFHPUST, BAHP, BHP, and AHP all showed a higher fluorescence peak at approximately 640 nm, which is similar to the natural phycocyanin. The results signified that phycocyanin was successfully expressed and could bind PCB to present optical activity. However, the peak of the fluorescence spectrum showed a certain degree of blue shift away from the natural phycocyanin (643 nm), which is probably attributable to the different structure of heterologous expression. In the chromophore-lyase absent strains, BAPH, BPH, and APH, phycocyanin or even individual subunit-β or subunit-αcould also bind to phycocyanobilin and exhibit autocatalytic activity. The color and fluorescence intensity of strain BAEFHPUST was the strongest, which is probably caused by the chromophore lyase promoting the binding between PCB and apo-phycocyanin. The recombinant strain HP also exhibited a light blue-green color, though it did not have apo-phycocyanin, perhaps owing to the fact that the strain could synthesize PCB. However, the color of HP was very light, and the fluorescence peak was very low and had a 5 nm red-shift to the natural phycocyanin, indicating that the fluorescence was not from the phycocyanin, but rather from the PCB.

Recently, Wu et al. (2016) reported that in *Arthrospira platensis* FACHB314, the chromophore lyases CpcS and CpcU can specifically link PCB to β-PC at Cys-82 [21]. Zhang et al. (2014) reported that a new bilin lyase CpcT from *Arthrospira platensis* FACHB314 can attach PCB to β-PC at Cys-153 [16]. Zhao et al. (2007) reported a near-universal lyase for Cys-84-binding sites in cyanobacterial phycobiliproteins [14]. To further explore the active site of apo-phycocyanin in *G. lemaneiformis*, recombinant strains BHP and AHP, as well as mutant strains B (82) HP, B (153) HP, B (82, 153) HP, and A (84) HP were constructed. Although the mutant strains B (82) HP, B (153) HP, and B (82,153) HP also showed blue-green color, the fluorescence intensities were weaker than that of the recombinant strain BHP (Figure 9). Similarly, the fluorescence intensity of A (84) HP was also weaker than the recombinant strain AHP. The fluorescence intensity of the mutant strains B (82) HP, B (153) HP, B (82, 153) HP, and A (84) HP was similar to that of the recombinant strain HP without apo-phycocyanin, which may indicate that the fluorescence was not from the phycocyanin, but instead from the PCB.

According to the experimental results of Western blotting, it was confirmed that phycocyanin was present in the mutant strains. A decrease in the fluorescence intensity of the mutant strains demonstrated that the change in the mutation site resulted in a decrease in the fluorescent activity of the phycocyanin. It was further demonstrated that C82 and C153 on the β subunit and C84 on the α subunit may be the active sites of apo-phycocyanin to bind PCB. These results are consistent with the results in cyanobacteria.

These results demonstrate that the assembly mechanism of phycocyanin in cyanobacteria is still applicable in red algae *G. lemaneiformis*. The active sites of apo-phycocyanin on α subunit in *G. lemaneiformis* may be Cys-84 and on β subunit may be Cys-82 and Cys-153.

## Figures and Tables

**Table 1:** Strains and plasmids used in this study.

Strain and Plasmid	Source	Application
asapMD19-T	TaKaRa (Dalian, China)	Cloning vector
pACYCDuet-1	Novagen (Germany)	Expression vector
pET24a	Novagen (Germany)	Expression vector
pET24a-*ho*-*pcyA*	Our laboratory	Express heme oxygenase and ferredoxin oxidoreductase (HO and PcyA)
pACYCDuet-*cpcBA*-*cpcEF*	Our laboratory	Expression of apo-phycocyanin and chromophore lyase (CpcE and CpcF)
pET24a-*ho*-*pcyA*-UST	Our laboratory	Express heme oxygenase, ferredoxin oxidoreductase and chromophore lyase (CpcU, CpcS and CpcT)

**Table 2:** Primers of *pcBA* gene.

Primer Names	Primer Sequences	Annealing Temperature (°C)
*pcB* primers	*pcB*-F:5′-GGATCCATGCTAGATGCATTTGCTAAAGTTGT-3′	57.8
	*pcB*-R:5′-GAGCTCTTATACTACTGCAACTGCTG-3′	55.7
*pcA* primers	*pcA*-F:5′-GGATCCATGAAAACACCTATAACAGA-3′	52.6
	*pcA*-R:5′-GAGCTCTTAAGTTAAAGCATTAATGGCATAGTCT-3′	56.7
*pcBA* primers	*pcB*-F:5′-GGATCCATGCTAGATGCATTTGCTAAAGTTGT-3′	57.8
	*pcA*-R:5′-GAGCTCTTAAGTTAAAGCATTAATGGCATAGTCT-3′	56.7

**Table 3:** Mutant primers for the genes *pcB* and *pcA*.

Primer Names	Primer Sequences	Annealing Temperature (°C)
*pcB* mutation primers 1	*pcB*(82)-F:5′-GCAGCTGCTTTAAGAGATATGGAGATTGTTTTAA-3′	56.7
	*pcB*(82)-R:5′-CATTCTACGACTCGTATATGCATTACCACCT-3′	57.7
*pcB* mutation primers 2	*pcB*(153)-F:5′-GGAGATGCTAGCTCATTAATCGCTGAATTAGGA-3′	59.1
	*pcB*(153)-R:5′-TAGAGTTACACCATTTGTATTATTAGCTAAACTTA-3′	53.3
*pcA* mutation primers	*pcA*(84)-F:5′-AAAAGCTGCACGTGA CATAGGATAT-3′	52.3
	*pcA*(84)-R:5′-GCTTTTCCTATTGCACTAGATG-3′	49.4

**Table 4:** Designation of the recombinant strains.

Names of the Transformed	Expression Vectors	*E. coli* Strains
Control	pACYCDuet-1	pET-24a (+)
BAHP	pACYCDuet-*pcBA*	pET24a-*ho*-*pcyA*
BHP	pACYCDuet-*pcB*	pET24a-*ho*-*pcyA*
B(82)HP	pACYCDuet-*pcB*(C82A)	pET24a-*ho*-*pcyA*
B(153)HP	pACYCDuet-*pcB*(C153A)	pET24a-*ho*-*pcyA*
B(82,153)HP	pACYCDuet-*pcB*(C82A,C153A)	pET24a-*ho*-*pcyA*
AHP	pACYCDuet-*pcA*	pET24a-*ho*-*pcyA*
A(84)HP	pACYCDuet-*pcA*(C84A)	pET24a-*ho*-*pcyA*
BAEFHPUST	pACYCDuet-*pcBA-cpcEF*	pET24a-*ho*-*pcyA-cpcUST*
